# Supplementary material for: Microtubules provide directional information for core PCP function
Source: eLife. 2014 Aug 14;3:e02893. doi: 10.7554/eLife.02893 (PMC4151085; doi:10.7554/eLife.02893)
Supplement: Supplementary file 1. — DOI: http://dx.doi.org/10.7554/eLife.02893.024 [file elife02893s001.pdf]

Supplementary file 1

S1 Mathematical model formulation

In this work, we consider a rectangular array of cells that forms a close-packed planar arrangement of regular hexagonally shaped cells. In addition, we assume the geometry remains fixed throughout the simulation.

We use Ordinary Differential Equations (ODEs) to model Planar Cell Polarity (PCP) signaling. This is justified by our previous PCP modeling work [S1, S2], which showed that diffusion rates of proteins and complexes in the Partial Differential Equation (PDE) model are either very large (all except DshFz) or very small (DshFz). Thus the diffusion terms in the PDE model can be replaced by their quasi-steady-state solutions to reduce the PDE model to an ODE model, in which proteins and complexes have uniform distributions over the region where the protein or complex is free to diffuse. Therefore, a single variable is used to represent the concentration of the protein or complex in that region. Reaction equations thus represent reactions over regions of the cell and affect the uniform concentration of the proteins and complexes within these regions. Three different types of regions were defined, creating three categories of proteins and complexes (Fig. M1). Proteins that are present inside the interior of the cell are represented by a single variable in each cell. A single variable for each cell is also used to represent the uniform concentration of proteins and complexes that diffuse freely throughout the cell membrane. Thus $[\mathrm{X}]_i$ represents the uniform concentration of protein or complex X in the i-th cell's interior or membrane. Complexes that form across a shared edge between two neighboring cells, for example, are represented by a separate variable for each edge of the cell. Thus $[\mathrm{X}]_{i,j}$ represents the uniform concentration of complex X at the j-th edge of the i-th cell.

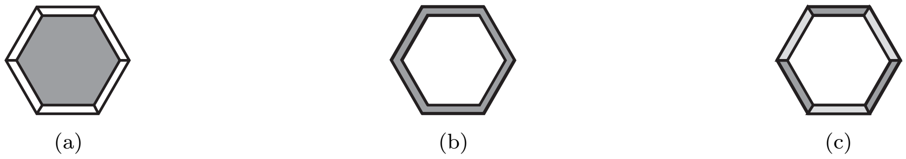

Figure M1: Cell regions represented in the model. (a) Cell interior. (b) Complete membrane. (c) Shared membranes.

S1.1 Ft/Ds/Fj global module

The mathematical model of the Ft/Ds/Fj module represents the interaction between Ft and Ds to form the protein complex FtDs [S3, S4]. More specifically, Ft on the membrane of one cell reacts with Ds on the membrane of a neighboring cell to form the complex FtDs. This reaction is regulated by Fj in two ways. Firstly, Fj increases the ability of Ft to bind Ds, thus promotes the formation of FtDs when Fj is upregulated in the Ft-expressing cell. Secondly, Fj reduces the ability of Ds to bind Ft and thus inhibits the formation of FtDs when Fj is upregulated in the Ds-expressing cell. Backward reaction separates FtDs complex back into Ft and Ds.

This is represented by the following reaction occurring at the cell edges.

$$\mathrm{Ft} + \mathrm{Ds}^{\dagger} \underset{\lambda_0}{\overset{BR_0}{\rightleftharpoons}} \mathrm{FtDs} \tag{S1}$$

where Fj regulation is introduced through

$$B = \exp\left(K_{\mathrm{Ft}}[\mathrm{Fj}] - K_{\mathrm{Ds}}[\mathrm{Fj}]^{\dagger}\right) \tag{S2}$$

R_0 is the forward reaction rate constant. λ_0 is the backward reaction rate constant. The effect of Fj regulating the formation of FtDs complex is modeled in Eq. S1 as a multiplier of the forward reaction rate. It is the exponential of the difference in Fj concentrations in the two neighboring cells scaled by constants of proportionality K_{Ft} and K_{Ds}. K_{Ft} represents the strength of Fj in increasing the ability of Ft to bind Ds, and likewise K_{Ds} represents the strength of Fj in reducing the ability of Ds to bind Ft. K_{Ft} and K_{Ds} were set to 10, so that Fj had equal effects on Ft and Ds. The daggered (†) variables indicate that the reaction occurs with the protein across the cell membrane in a neighboring cell. For notation purposes, we chose to associate the complexes spanning multiple cells with the source cell for Ft. The products from the backward reaction of Eq. S1 are located in their original cells so that the total amount of each protein in a cell is conserved.

Ft, Ds and Fj are assumed to diffuse freely in the cell membrane. FtDs is restricted to diffuse in the cell edge common to the cells involved in the formation of the complex.

The net forward reaction rate for Eq. S1 occurring at the j-th edge of the i-th cell is

$$P_{0,i,j} = BR_0[\mathrm{Ft}]_i[\mathrm{Ds}]_i^\dagger - \lambda_0[\mathrm{FtDs}]_{i,j}. \tag{S3}$$

The set of ODEs that govern the rates of change for each protein and complex concentration is given by

$$\frac{d[\mathrm{Ft}]_i}{dt} = \frac{1}{N}\sum_{j=1}^{N}\left(-P_{0,i,j}\right) \tag{S4}$$

$$\frac{d[\mathrm{Ds}]_i}{dt} = \frac{1}{N}\sum_{j=1}^{N}\left(-P_{0,i^\dagger,j^\dagger}\right) \tag{S5}$$

$$\frac{d[\mathrm{FtDs}]_{i,j}}{dt} = P_{0,i,j} \tag{S6}$$

where $N = 6$ is the total number of edges of a hexagonal cell. Note Fj is neither a reactant nor a product in Eq. S1, thus its concentration does not change with time.

S1.2 Microtubule (MT) network

The mathematical model for the MT network assumes MT pattern is determined by Ft distribution, the more asymmetric the distribution of Ft is within a cell, the more polarized the MT pattern is (i.e. more aligned with proximal-distal axis). Free Ft is uniformly distributed around the cell membrane, thus the asymmetry in Ft distribution and consequently the number of MTs between any two cell edges only depends on FtDs concentrations at cell edges. Therefore the number of MTs between two edges j and k of the i-th cell, with plus-ends at the k-th edge is proportional to

$$M_{i,j\to k} = \rho_i K_u + (1-\rho_i)\,K_a\left|[\mathrm{FtDs}]_{i,j} - [\mathrm{FtDs}]_{i,k}\right| \times \exp\left(K_d\left([\mathrm{FtDs}]_{i,j} - [\mathrm{FtDs}]_{i,k}\right)\right) \tag{S7}$$

where $\rho_i = \exp\left(-K_\rho||\vec{\mathrm{Ft}}_i||^2\right)$ and K_a, K_d, K_u and K_ρ are constants.

$M_{i,j\to k}$ is the convex combination of two terms. The mixing proportions of these two terms is controlled by $\rho_i \in (0, 1]$, which captures the degree of asymmetric Ft distribution in cell i. $\rho_i = 1$ represents uniform distribution of Ft. As $\rho_i \to 0$, Ft distribution becomes increasingly asymmetric. We define $\vec{\mathrm{Ft}}_i := \sum_{j=1}^{N}([\mathrm{FtDs}]_{i,j}\vec{e_j})$ as the vector sum of Ft concentration within cell i, and $\vec{e_j}$ represents the unit vector pointing from the cell center towards the mid-point of the j-th edge.

When Ft distribution is uniform, $||\vec{\mathrm{Ft}}_i||$ is equal to zero, thus $\rho_i = 1$ and $M_{i,j\to k} = K_u$ between all edges j and k, representing randomized MT orientation. A more asymmetric Ft distribution leads to greater values of $||\vec{\mathrm{Ft}}_i||$, which corresponds to smaller values of ρ_i. Thus Eq. S7 becomes increasingly dominated by the second term: $K_a\left|[\mathrm{FtDs}]_{i,j} - [\mathrm{FtDs}]_{i,k}\right|\exp\left(K_d\left([\mathrm{FtDs}]_{i,j} - [\mathrm{FtDs}]_{i,k}\right)\right)$. The norm $\left|[\mathrm{FtDs}]_{i,j} - [\mathrm{FtDs}]_{i,k}\right|$ models the observation that most MTs run between proximal and distal edges and fewer MTs are observed between anterior and posterior edges. The exponential term models the observation that there is a small but significant surplus of MTs with distally pointed plus-ends, than those with proximally pointed plus-ends [S5].

The last example in Fig. 5, of the main text, shows simulation of an unbiased but oriented MT arrangement in the distal region. For this case only, a fixed MT pattern is defined (Fig. M2). For cells located in the proximal region in this example, the parameters $\alpha_{1,i}$, $\alpha_{2,i}$, β_i and γ_i are chosen such that both the total

number of MTs and the resulting MT arrangement in the proximal, closely matches that of the previous example where the Ds concentration profile is identical, albeit MT orientation is randomized in the distal region where there is zero level of Ds. In the distal region, $\alpha_{1,i}$ and $\alpha_{2,i}$ values are identical to those in the proximal region, thus the resulting MT arrangement maintains a similar asymmetric distribution. β_i is set to 1 for cells in the distal so that there is no plus-end bias. γ_i is chosen to ensure the total number of MTs remains the same in both proximal and distal cells.

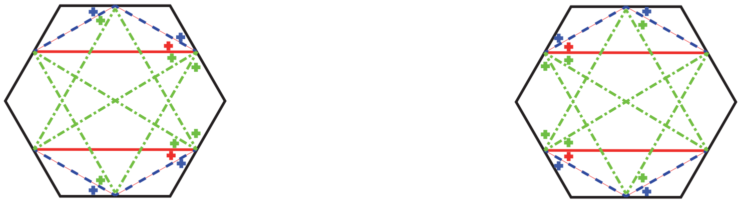

(a) (b)

Figure M2: Fixed MT pattern. (a) MTs with plus-end towards distal side. Number of MTs represented by red solid lines is proportional to γ_i. Number of MTs represented by blue dashed lines is proportional to $\alpha_{1,i}\gamma_i$. Number of MTs with green dash-dotted lines is proportional to $\alpha_{2,i}\gamma_i$. (b) MTs with plus-end towards proximal side. Number of MTs represented by red solid lines is proportional to $\beta_i\gamma_i$. Number of MTs represented by blue dashed lines is proportional to $\beta_i\alpha_{1,i}\gamma_i$. Number of MTs with green dash-dotted lines is proportional to $\beta_i\alpha_{2,i}\gamma_i$. $0 \leq \alpha_{2,i} \leq \alpha_{1,i} \leq 1$. $0 \leq \beta_i \leq 1$.

S1.3 Core module

The mathematical model of the core module represents a network of five proteins, Dsh, Fz, Pk, Vang and an unknown membrane protein X. The essential logic of the feedback loop of the core module is achieved through protein interactions to form protein complexes and MT facilitated directed trafficking of specific complexes. For example, Dsh interacts with Fz to form a DshFz complex, Vang interacts with Pk to form VangPk, and X interacts with Dsh to form XDsh. Protein X is introduced to allow Dsh to associate with the cell cortex, even in the absence of other core proteins, as is experimentally observed. X may not be a real protein, as Dsh is thought to associate with membrane phospholipids electrostatically [S6]. For reactions between cells, Fz on the membrane of one cell reacts with Vang on the membrane of an adjacent cell to form a complex FzVang. FzVang can further react with Dsh or Pk to form larger complexes. Backward reactions separate the complexes back into their constituent proteins and locate them in their original cells.

The seven protein complexes included in this model are XDsh, DshFz, VangPk, FzVang, DshFzVang, FzVangPk and DshFzVangPk. The last four of these complexes are formed across the cell membrane shared by two neighboring cells. We assume XDsh and DshFz undergo directed vesicle trafficking. Dsh and Pk are assumed to diffuse freely within the cell interior. X, Fz, Vang and VangPk diffuse around the whole cell membrane. The four complexes that form between neighboring cells are restricted to diffuse in the cell edge common to the cells involved in the formation of the complex. Though the complex DshFz is not formed across a shared cell edge, our previous modeling work on PCP signaling [S1, S2] suggested that diffusion rate of DshFz is likely to be very small. Consequently in this model, we follow the assumption in our previous models: diffusion of DshFz is restricted to each cell edge rather than the whole cell membrane. In addition, we assume XDsh is similarly restricted to diffuse freely only at each cell edge.

The model is represented by the following fourteen reactions occurring at the cell edges.

$$\mathrm{Dsh} + \mathrm{Fz} \underset{\lambda_1}{\overset{R_1}{\rightleftharpoons}} \mathrm{DshFz} \tag{S8}$$

$$\mathrm{Fz}^{\dagger} + \mathrm{Vang} \underset{\lambda_2}{\overset{R_2}{\rightleftharpoons}} \mathrm{FzVang} \tag{S9}$$

$$\mathrm{Vang} + \mathrm{Pk} \underset{\lambda_3}{\overset{R_3}{\rightleftharpoons}} \mathrm{VangPk} \tag{S10}$$

$$\mathrm{DshFz}^\dagger + \mathrm{Vang} \underset{\lambda_4}{\overset{R_4}{\rightleftharpoons}} \mathrm{DshFzVang} \tag{S11}$$

$$\mathrm{Dsh}^\dagger + \mathrm{FzVang} \underset{\lambda_5}{\overset{R_5}{\rightleftharpoons}} \mathrm{DshFzVang} \tag{S12}$$

$$\mathrm{Fz}^\dagger + \mathrm{VangPk} \underset{\lambda_6}{\overset{R_6}{\rightleftharpoons}} \mathrm{FzVangPk} \tag{S13}$$

$$\mathrm{FzVang} + \mathrm{Pk} \underset{\lambda_7}{\overset{R_7}{\rightleftharpoons}} \mathrm{FzVangPk} \tag{S14}$$

$$\mathrm{Dsh}^\dagger + \mathrm{FzVangPk} \underset{\lambda_8}{\overset{R_8}{\rightleftharpoons}} \mathrm{DshFzVangPk} \tag{S15}$$

$$\mathrm{DshFz}^\dagger + \mathrm{VangPk} \underset{\lambda_9}{\overset{R_9}{\rightleftharpoons}} \mathrm{DshFzVangPk} \tag{S16}$$

$$\mathrm{DshFzVang} + \mathrm{Pk} \underset{\lambda_{10}}{\overset{R_{10}}{\rightleftharpoons}} \mathrm{DshFzVangPk} \tag{S17}$$

$$\mathrm{X} + \mathrm{Dsh} \underset{\lambda_{11}}{\overset{R_{11}}{\rightleftharpoons}} \mathrm{XDsh} \tag{S18}$$

$$\mathrm{XDsh} + \mathrm{Fz} \underset{\lambda_{12}}{\overset{R_{12}}{\rightleftharpoons}} \mathrm{X} + \mathrm{DshFz} \tag{S19}$$

$$\mathrm{XDsh}^\dagger + \mathrm{FzVang} \underset{\lambda_{13}}{\overset{R_{13}}{\rightleftharpoons}} \mathrm{X}^\dagger + \mathrm{DshFzVang} \tag{S20}$$

$$\mathrm{XDsh}^\dagger + \mathrm{FzVangPk} \underset{\lambda_{14}}{\overset{R_{14}}{\rightleftharpoons}} \mathrm{X}^\dagger + \mathrm{DshFzVangPk} \tag{S21}$$

$R_1, R_2, \ldots, R_{14}$ are forward reaction rates. $\lambda_1, \lambda_2, \ldots, \lambda_{14}$ are backward reaction rates. As in Section S1.1, the daggered variables indicate that reactions occur between proteins from two adjacent cells across a shared cell edge. Without loss of generality, we chose to associate the complexes spanning multiple cells with the source cell for Vang.

The net forward reaction rates for Eqs. S8 to S21 at each cell edge are denoted by $P_{1,i,j}$ through $P_{14,i,j}$. They represent the difference between the forward and backward reactions.

$$P_{1,i,j} = R_1[\mathrm{Ds}]_i[\mathrm{Fz}]_i - \lambda_1[\mathrm{DshFz}]_{i,j} \tag{S22}$$

$$P_{2,i,j} = R_2[\mathrm{Fz}]^\dagger_{i^\dagger}[\mathrm{Vang}]_i - \lambda_2[\mathrm{FzVang}]_{i,j} \tag{S23}$$

$$P_{3,i} = R_3[\mathrm{Vang}]_i[\mathrm{Pk}]_i - \lambda_3[\mathrm{VangPk}]_i \tag{S24}$$

$$P_{4,i,j} = R_4[\mathrm{DshFz}]^\dagger_{i^\dagger,j^\dagger}[\mathrm{Vang}]_i - \lambda_4[\mathrm{DshFzVang}]_{i,j} \tag{S25}$$

$$P_{5,i,j} = R_5[\mathrm{Dsh}]^\dagger_{i^\dagger}[\mathrm{FzVang}]_{i,j} - \lambda_5[\mathrm{DshFzVang}]_{i,j} \tag{S26}$$

$$P_{6,i,j} = R_6[\mathrm{Fz}]^\dagger_{i^\dagger}[\mathrm{VangPk}]_i - \lambda_6[\mathrm{FzVangPk}]_{i,j} \tag{S27}$$

$$P_{7,i,j} = R_7[\mathrm{FzVang}]_{i,j}[\mathrm{Pk}]_i - \lambda_7[\mathrm{FzVangPk}]_{i,j} \tag{S28}$$

$$P_{8,i,j} = R_8[\mathrm{Dsh}]^\dagger_{i^\dagger}[\mathrm{FzVangPk}]_{i,j} - \lambda_8[\mathrm{DshFzVangPk}]_{i,j} \tag{S29}$$

$$P_{9,i,j} = R_9[\text{DshFz}]^{\dagger}_{i^\dagger,j^\dagger}[\text{VangPk}]_i - \lambda_9[\text{DshFzVangPk}]_{i,j} \tag{S30}$$

$$P_{10,i,j} = R_{10}[\text{DshFzVang}]_{i,j}[\text{Pk}]_i - \lambda_{10}[\text{DshFzVangPk}]_{i,j} \tag{S31}$$

$$P_{11,i,j} = R_{11}[\text{X}]_i[\text{Dsh}]_i - \lambda_{11}[\text{XDsh}]_{i,j} \tag{S32}$$

$$P_{12,i,j} = R_{12}[\text{XDsh}]_{i,j}[\text{Fz}]_i - \lambda_{12}[\text{X}]_i[\text{DshFz}]_{i,j} \tag{S33}$$

$$P_{13,i,j} = R_{13}[\text{XDsh}]^{\dagger}_{i^\dagger,j^\dagger}[\text{FzVang}]_{i,j} - \lambda_{13}[\text{X}]^{\dagger}_{i^\dagger}[\text{DshFzVang}]_{i,j} \tag{S34}$$

$$P_{14,i,j} = R_{14}[\text{XDsh}]^{\dagger}_{i^\dagger,j^\dagger}[\text{FzVangPk}]_{i,j} - \lambda_{14}[\text{X}]^{\dagger}_{i^\dagger}[\text{DshFzVangPk}]_{i,j} \tag{S35}$$

The set of ODEs that govern the time rates of change for each protein and complex concentration is given by

$$\frac{d[\text{Dsh}]_i}{dt} = \frac{1}{C}\sum_{j=1}^{N}\left(-P_{1,i,j} - P_{5,i^\dagger,j^\dagger} - P_{8,i^\dagger,j^\dagger} - P_{11,i,j}\right) \tag{S36}$$

$$\frac{d[\text{Pk}]_i}{dt} = \frac{1}{C}\left(\sum_{j=1}^{N}\left(-P_{7,i,j} - P_{10,i,j}\right) - N \times P_{3,i}\right) \tag{S37}$$

$$\frac{d[\text{X}]_i}{dt} = \frac{1}{N}\sum_{j=1}^{N}\left(-P_{11,i,j} + P_{12,i,j} + P_{13,i^\dagger,j^\dagger} + P_{14,i^\dagger,j^\dagger}\right) \tag{S38}$$

$$\frac{d[\text{Fz}]_i}{dt} = \frac{1}{N}\sum_{j=1}^{N}\left(-P_{1,i,j} - P_{2,i^\dagger,j^\dagger} - P_{6,i^\dagger,j^\dagger} - P_{12,i,j}\right) \tag{S39}$$

$$\frac{d[\text{Vang}]_i}{dt} = \frac{1}{N}\left(\sum_{j=1}^{N}\left(-P_{2,i,j} - P_{4,i,j}\right) - N \times P_{3,i}\right) \tag{S40}$$

$$\frac{d[\text{VangPk}]_i}{dt} = \frac{1}{N}\left(\sum_{j=1}^{N}\left(-P_{6,i,j} - P_{9,i,j}\right) + N \times P_{3,i}\right) \tag{S41}$$

$$\frac{d[\text{FzVang}]_{i,j}}{dt} = P_{2,i,j} - P_{5,i,j} - P_{7,i,j} - P_{13,i,j} \tag{S42}$$

$$\frac{d[\text{DshFzVang}]_{i,j}}{dt} = P_{4,i,j} + P_{5,i,j} - P_{10,i,j} + P_{13,i,j} \tag{S43}$$

$$\frac{d[\text{FzVangPk}]_{i,j}}{dt} = P_{6,i,j} + P_{7,i,j} - P_{8,i,j} - P_{14,i,j} \tag{S44}$$

$$\frac{d[\text{DshFzVangPk}]_{i,j}}{dt} = P_{8,i,j} + P_{9,i,j} + P_{10,i,j} + P_{14,i,j} \tag{S45}$$

$$\frac{d[\text{XDsh}]_{i,j}}{dt} = P_{11,i,j} - P_{12,i,j} - P_{13,i^\dagger,j^\dagger} - P_{14,i^\dagger,j^\dagger} + \sum_{k=1}^{N}\left(\delta_{\text{XDsh}_{MT},i,k\to j} - \delta_{\text{XDsh}_{MT},i,j\to k}\right) \tag{S46}$$

$$\frac{d[\text{DshFz}]_{i,j}}{dt} = P_{1,i,j} - P_{4,i^\dagger,j^\dagger} - P_{9,i^\dagger,j^\dagger} + P_{12,i,j} + \sum_{k=1}^{N}\left(\delta_{\text{DshFz}_{MT},i,k\to j} - \delta_{\text{DshFz}_{MT},i,j\to k}\right). \tag{S47}$$

The last two terms in Eqs. S46 and S47 represent the change in XDsh and DshFz concentration due to directed vesicle trafficking. $\delta_{\bullet_{MT},i,k\to j}$ represents increase in the concentration of XDsh or DshFz at edge j

due to vesicle trafficking of the protein complex from another edge k to edge j, whereas $\delta_{\bullet_{MT},i,j\to k}$ represents decrease in the concentration of XDsh or DshFz at edge j due to vesicle trafficking taking protein complex away from edge j and placing it onto another edge k. Directed vesicle trafficking is modeled as a convection process

$$\delta_{\bullet_{MT},i,j\to k} = M_{i,j\to k} A_{i,j} [\bullet]_{i,j} \tag{S48}$$

where $M_{i,j\to k}$ is as defined in Section S1.2 and is proportional to the number of MTs in cell i between the edges j and k with plus-ends located at edge k. The effect of Pk and Vang in inhibiting the accumulation of Dsh at the membrane is modeled as a promotion in the rate of directed trafficking of Dsh-containing vesicles away from the edge where they co-locate. We model this promotion as being proportional to the local concentration of Pk and Vang at the cell edge raised to the exponent K_p, where K_p models non-linearity in the inhibitory effects of Pk and Vang.

$$\begin{aligned} A_{i,j} = ([\mathrm{Pk}]_i + [\mathrm{Vang}]_i + [\mathrm{VangPk}]_i + [\mathrm{FzVang}]_{i,j} \\ + [\mathrm{DshFzVang}]_{i,j} + [\mathrm{FzVangPk}]_{i,j} + [\mathrm{DshFzVangPk}]_{i,j})^{K_p}. \end{aligned} \tag{S49}$$

S2 Numerical methods

S2.1 Initial conditions

Initial conditions for our ODE model represent initial concentrations of proteins and complexes. Initial concentrations of all complexes are set to zero. For the global module, the concentrations of Fj and Ds are uniform within each cell membrane and their proximal-distal concentration profiles are user-defined in each simulation to test the effect of different expression profiles on the MT network arrangement and the resulting PCP signal. Initial concentration of Ft is uniform throughout the tissue and is specified as a model parameter. For the core module, initial concentrations of all proteins Dsh, Fz, Vang, Pk and X are also uniform and specified as input parameters.

S2.2 Boundary conditions

Periodic boundary conditions are used parallel to the anterior-posterior axis, thus we are effectively simulating a two-dimensional array of hexagonal cells extending infinitely along the anterior-posterior axis by repeated tiling of the smaller array of cells.

Periodic boundary conditions could not be applied to the proximal-distal axis due to lack of symmetry in Ds and Fj expression profiles along this axis. Therefore, a set of free boundary conditions, similar to that used in our previous ODE model [S2], was defined that extrapolated data for boundary cells from the nearest neighboring interior cell. Boundary cells are defined as cell with at least one edge not shared with a neighboring cell and distance between cells are defined as the distance between the center of the cells. The concentration values of proteins and complexes that are free to diffuse throughout the interior or membrane of the cell (Ds, Ft, X, Dsh, Fz, Vang, Pk and VangPk) are copied from the nearest interior cell to the boundary cell. The concentration of complexes that are restricted to an edge of a boundary cell, are computed by solving the ODEs for these values in the same way as for other interior edges. No boundary condition is necessary for the unshared edges because these values are not used elsewhere in the simulation.

S2.3 ODE solver

The set of ODEs is solved in MATLAB using function *ode23*, with initial and boundary conditions described in Sections S2.1 and S2.2. The Ft/Ds/Fj module is simulated first until steady state is reached. The steady state FtDs distribution is used to compute MT network arrangement, which is then used as an input in the simulation of the core module to compute final distribution of PCP core proteins and complexes.

S2.4 Hair growth direction prediction

Hair growth direction is determined using steady state protein concentrations at the end of simulation. We follow our previous model's assumption and use Dsh localization to predict hair growth direction [S1, S2]. In other words, the hair growth direction in each cell is computed as the direction corresponding to the vector sum of Dsh concentrations at the cell edges. If Dsh was not polarized above a set threshold (i.e. the magnitude of the vector sum of Dsh concentrations in a given cell is not above a specified threshold), then the hair is assigned to the center of the cell.

S3 Parameter selection

The unspecified model parameters are the initial concentrations for proteins Ft, Dsh, Fz, Vang, Pk and X (denoted $[\mathrm{Ft}]_0$, $[\mathrm{Dsh}]_0$, $[\mathrm{Fz}]_0$, $[\mathrm{Vang}]_0$, $[\mathrm{Pk}]_0$ and $[\mathrm{X}]_0$), the forward and backward reaction rate constants R_0 through R_{14} and λ_0 through λ_{14}, and constants K_{Ft}, K_{Ds}, K_u, K_a, K_d, K_ρ and K_p. We assume Fj's effect to increase the ability of Ft to bind with Ds is equal to its effect to decrease the ability of Ds to bind with Ft, thus reducing the number of parameters by setting $K_{Ft} = K_{Ds}$. Protein and complex concentrations are relative to each other and their unit of measurement is unspecified, thus without loss of generality, we fixed $[\mathrm{Dsh}]_0$ to 1, hence further reducing the number of parameters. Initial concentrations of core module proteins $[\mathrm{Fz}]_0$, $[\mathrm{Vang}]_0$, $[\mathrm{Pk}]_0$ and the reaction rate constants R_1 through R_{10} and λ_1 through λ_{10} are taken from our previous mathematical model for the core PCP signaling module [S2]. Remaining parameters are chosen so that the simulation would reproduce qualitative features of the experimentally observed PCP phenotypes, such as the presence of (or the lack of) domineering non-autonomy in different clones (Fig. S5).

References

S1. Amonlirdviman, K., Khare, N.A., Tree, D.R., Chen, W.S., Axelrod, J.D., and Tomlin, C.J. (2005). Mathematical modeling of planar cell polarity to understand domineering nonautonomy. Science *307*, 423-426.

S2. Raffard, R., Amonlirdviman, K., Axelrod, J.D., and Tomlin, C.J. (2008). An adjoint-based parameter identification algorithm applied to planar cell polarity signaling. IEEE Transactions on Automatic Control *53*, 109-121.

S3. Matakatsu, H., and Blair, S.S. (2004). Interactions between Fat and Dachsous and the regulation of planar cell polarity in the Drosophila wing. Development *131*, 3785-3794.

S4. Simon, M.A., Xu, A., Ishikawa, H.O., and Irvine, K.D. (2010). Modulation of Fat-Dachsous binding by the cadherin domain kinase Four-jointed. Curr Biol *20*, 811-817.

S5. Harumoto, T., Ito, M., Shimada, Y., Kobayashi, T.J., Ueda, H.R., Lu, B., and Uemura, T. (2010). Atypical cadherins Dachsous and Fat control dynamics of noncentrosomal microtubules in planar cell polarity. Dev Cell *19*, 389-401.

S6. Simons, M., Gault, W.J., Gotthardt, D., Rohatgi, R., Klein, T.J., Shao, Y., Lee, H.J., Wu, A.L., Fang, Y., Satlin, L.M., et al. (2009). Electrochemical cues regulate assembly of the Frizzled/Dishevelled complex at the plasma membrane during planar epithelial polarization. Nat Cell Biol *11*, 286-294.
